# A Boron-Based Topical Strategy for Enhancing Flap Survival: Mechanistic Insights Through Proteomic Analysis

**DOI:** 10.3390/biomimetics10110741

**Published:** 2025-11-05

**Authors:** Cafer Yildirim, Merve Gulsen Bal Albayrak, Sevinc Yanar, Nihal Kayir, Ayse Hande Yozgat, Sevim Aydin, Fikrettin Şahin

**Affiliations:** 1 Department of Basic Medical Sciences, Faculty of Dentistry, Ankara University, Ankara 06560, Turkey; cfryildirim@ankara.edu.tr; 2 Department of Medical Biology, Faculty of Medicine, Kocaeli University, Kocaeli 41001, Turkey; 3 Department of Molecular Gastroenterology and Hepatology, Gastroenterology and Hepatology Institute, Kocaeli University, Kocaeli 41001, Turkey; 4 Department of Medical Biology, Faculty of Medicine, Ankara Yildirim Beyazit University, Ankara 06560, Turkey; sevincyanar@aybu.edu.tr; 5 Department of Medical Pharmacology, Faculty of Medicine, Medipol University, İstanbul 34820, Turkey; nihalkayirr@gmail.com; 6 Department of Histology and Embryology, Faculty of Medicine, Ankara University, Ankara 06560, Turkey; hyozgat@ankara.edu.tr (A.H.Y.); sevimaydin@ankara.edu.tr (S.A.); 7 Department of Genetics and Bioengineering, Faculty of Engineering, Yeditepe University, Istanbul 34758, Turkey; fsahin@yeditepe.edu.tr

**Keywords:** flap wound model, boron, sodium pentaborate pentahydrate (SPP), Dermobor^®^, wound healing, proteomics, reconstructive surgery

## Abstract

Flap viability remains a major challenge in reconstructive surgery due to ischemia–reperfusion injury, excessive inflammation, and impaired tissue regeneration. Boron, a trace element with pro-healing and anti-inflammatory properties, has shown therapeutic promise in various wound models; however, its role in flap healing remains unclear. In this study, we aimed to evaluate the therapeutic potential of sodium pentaborate pentahydrate (SPP)-containing hydrogel, a boron compound we developed, for enhancing flap survival and tissue repair. A dorsal random-pattern flap model was established in male Wistar rats, which were treated topically with an SPP-containing formulation twice daily for seven days. Histological changes were evaluated using hematoxylin–eosin and Masson’s trichrome staining, and proteomic alterations were analyzed using label-free nanoLC-MS/MS followed by bioinformatics analysis. The treatment significantly improved flap survival (*p* < 0.0001), enhanced granulation tissue formation, promoted organized collagen deposition, and reduced inflammatory infiltration. Proteomic profiling identified 179 differentially expressed proteins, with 14 upregulated and 165 downregulated. Upregulated proteins were enriched in pathways related to complement activation, antioxidant defense, and extracellular matrix remodeling, whereas downregulated proteins were associated with immune overactivation, cellular stress, and senescence, indicating a shift toward regulated inflammation and tissue homeostasis. To our knowledge, this is the first study to demonstrate that an SPP-containing hydrogel promotes flap healing by supporting vascularization, modulating immune responses, and enhancing extracellular matrix remodeling. These findings highlight SPP as a promising therapeutic strategy for improving flap viability in reconstructive surgery.

## 1. Introduction

Flap surgery plays a pivotal role in reconstructive procedures, enabling the transfer of vascularized tissue from donor to recipient sites to repair complex defects resulting from congenital anomalies, trauma, burns, or oncologic resections. Despite their advantages in wound coverage and reducing infection risk, flap viability remains challenged by ischemia–reperfusion injury, microcirculatory failure, and distal necrosis, which occur in up to 20–80% of experimental models and 10–25% of clinical cases [1,2]. These issues are marked by oxidative stress, inflammation, impaired angiogenesis, and inadequate extracellular matrix (ECM) formation, leading to partial flap loss and adverse aesthetic and functional outcomes [3,4]. 

Preclinical research on random-pattern flap models, including the widely used ischemia–reperfusion model described by Ballestín et al., has identified key mechanisms underlying flap failure, such as inflammation-driven edema and reactive oxygen species (ROS)-mediated tissue damage [5]. Interventions explored in these models include vascular endothelial growth factor (VEGF) gene therapy, deferoxamine, stem cell-derived exosomes, antioxidants, and vasodilators, all aimed at promoting neovascularization, mitigating oxidative stress, and preserving ECM structure [6,7,8]. For instance, Wang et al. reported that deferoxamine significantly enhances HIF-1α and VEGF expression, thereby improving microvascular density and flap survival [6]. Despite such progress, the clinical translation of advanced biologicals and biomaterials, including growth factor-releasing scaffolds, remains limited, largely due to high costs, delivery complexities, and the risk of systemic side effects [9].

Bioinspired design principles mirror the structure–function relationships of healing tissues and guide engineered solutions in tissue engineering and biomaterials. Within this biomimetics framework, ECM-mimetic hydrogels that provide appropriate hydration, viscoelasticity, and ligand presentation can help coordinate cell migration and neovascularization [10]. In parallel, biologically relevant cues, including trace element signaling, offer nature-derived ways to modulate inflammation and angiogenesis. Among these cues, boron has attracted growing attention as a modulator of inflammatory and angiogenic responses [11].

Building on this rationale, recent studies indicate that boron can support wound repair by enhancing fibroblast activity, collagen organization, antimicrobial defense, and pro-angiogenic signaling [12]. In chronic and burn wound models, boron-containing hydrogels have shown notable effects such as enhancing fibroblast activity, promoting VEGF expression, and reducing local inflammation, thereby accelerating wound closure [13]. For example, carbopol and pluronic gel formulations enriched with boron improved re-epithelialization and angiogenesis in diabetic and burn wound animal models [14]. Recent advances include stem-cell-loaded boron-enriched scaffolds, which have demonstrated improved epithelialization, collagen deposition, and angiogenesis in full-thickness burn wounds [15]. Additionally, lipoic acid-conjugated boron nanoparticles and zinc borate complexes have shown potential in enhancing fibroblast proliferation, collagen formation, and bacterial clearance, suggesting broader utility across wound types [16,17]. Furthermore, quercetin–boronic acid complexes have been investigated for diabetic foot ulcers and were found to significantly increase re-epithelialization, angiogenesis, and fibroblast activity compared to conventional treatments [18]. Nano-lanthanide borates and boron-based biohybrid films have also demonstrated pro-angiogenic and antioxidant properties, further supporting boron’s versatility in tissue-healing applications [19].

Despite these promising results, boron-containing hydrogels have not been previously evaluated in flap models. This represents a critical knowledge gap given the complexity of flap ischemia and reperfusion dynamics [4]. Our study therefore seeks to translate boron’s known benefits to a clinically relevant flap wound platform. Using a refined rat flap model, we applied a hydrogel containing sodium pentaborate pentahydrate (SPP) and evaluated its effect on healing through histological and proteomic analyses. The results confirmed enhanced angiogenesis, reduced inflammation, improved extracellular matrix organization, and balanced molecular signaling, demonstrating the versatile advantages of this formulation for reconstructive flap applications.

## 2. Materials and Methods

### 2.1. Preparation of the Gel

The base hydrogel was prepared following the previously described Dermobor^®^ formulation (Bi7Tech, Istanbul, Turkey) comprising sodium pentaborate pentahydrate (SPP) (produced in Fikrettin Sahin’s Laboratuary), Carbopol Ultrez-21 (Lubrizol, Wickliffe, OH, USA), and Pluronic copolymers (Sigma-Aldrich, St. Louis, MO, USA) [13,14]. Carbopol-Ultrez-21 was dispersed at 1% (*w*/*v*) in distilled water and neutralized with 1 M NaOH (Merck, Darmstadt, Germany) to adjust pH to 6.5–7.0. Subsequently, SPP (3% *w*/*v*), Pluronic F68 and F127 (2% *w*/*v* each), and here additionally potassium chloride (KCl (Sigma-Aldrich, St. Louis, MO, USA), 1% *w*/*w*) were incorporated into the blank gel. SPP served as the active therapeutic agent; Carbopol provided viscosity and tissue adherence, Pluronic F68/F127 supplied thermoresponsive gelation and residence time. Inverted-tube gelation has been repeatedly demonstrated for Pluronic-based thermogels [20,21]. KCl adjusted ionic strength. Additionally, K+ channel inhibition by KCl has been shown to enhance cutaneous wound closure in vitro and in vivo [22], supporting its role as a functional excipient. No growth factors, antibiotics, or other bioactive agents were included. The mixture was then cooled at 4 °C until fully dissolved (approximately 24 h) and deaerated prior to use. The addition of KCl yields the novel formulation, named Dermobor Plus (DBP).

The hydrogel was visually homogeneous and exhibited thermoresponsive behavior, being low-viscosity at 4 °C for ease of preparation and application and forming a semisolid at physiological temperature to support residence time. The material was readily spreadable for topical use. Selection of the SPP dose (3% *w*/*v*) was informed by prior SPP hydrogel studies demonstrating accelerated wound closure and improved histology in rodents, as well as by clinical trials reporting benefit with a 3% sodium pentaborate gel [23,24]. Accordingly, efficacy in the ischemic flap model was evaluated using a single dose, rather than a dose-finding study being performed.

### 2.2. Animals and Experimental Design

This study was conducted in the Experimental Research Laboratory of Ankara University Faculty of Medicine with the approval decision of Ankara University Animal Experiments Local Ethics Committee (2024-20-179). Sixteen male Wistar rats (Kobay A.Ş., Ankara, Turkey), aged 8 weeks and weighing between 250–300 g, were used. Prior to the experiment, animals were acclimatized to the laboratory environment for one week. They were housed under controlled environmental conditions with a 12-h light/dark cycle, stable temperature (21 ± 1 °C), and humidity (75 ± 5%), with ad libitum access to tap water and standard rat chow. Animals were monitored daily, and any notable changes in appearance or behavior were recorded. No dietary or water restrictions were applied.

The rats were randomly assigned to two groups (*n* = 8 per group): control and DBP-treated. All animals were age- and weight-matched male Wistar rats, housed under identical environmental and nutritional conditions, and underwent surgery performed by the same trained team using standardized procedures and instrumentation. Rats were given general anesthesia by intramuscular injection of a combination of 5 mg/kg xylazine hydrochloride (Bayer, Leverkusen, Germany) and 50 mg/kg ketamine hydrochloride (Pfizer, New York, NY, USA). After anesthesia, the dorsal skin of the rats was shaved and disinfected, and a 3 × 6 cm Mc-Farlane-type dorsal flaps were created on the back of each animal. The elevated flaps were immediately sutured back into their original positions. No treatment was applied in the control group. In the DBP-treated group, the hydrogel was topically applied to the flap site twice daily for 7 consecutive days. At the end of the treatment period, all animals were sacrificed, and samples were taken from the flap regions within surgical margins. The harvested tissues were separated for downstream analyses, with samples for histopathological evaluation fixed in 10% neutral-buffered formalin, and those intended for proteomic analysis snap-frozen in liquid nitrogen and stored at −80 °C until processing.

### 2.3. Tissue Collection and Histological Analysis

Skin samples obtained from each group were taken from the periphery of the wound seven days after wound creation, as well as from normal skin and DBP-treated skin areas. The samples were fixed in 10% buffered formalin, then subjected to standard dehydration protocol and embedded in paraffin blocks. Sections of 5 µm thickness were prepared from the paraffin blocks and stained with haematoxylin-eosin (H&E) and Masson’s Trichrome (MT) stains (Bio-Optica, Milan, Italy). Histological changes associated with wound healing in each specimen were examined and photographed using a light microscope (Zeiss Axio Scope A1, Oberkochen, Germany). The evaluation was performed by a sequential semi-quantitative scoring system based on four parameters: amount of granulation tissue, inflammatory cell infiltration, orientation of collagen fibres and collagen pattern. This scoring method was previously developed by Sultana and Santos and is presented in Table 1 [25,26]. All histopathological analyses were performed by two independent histologists in accordance with the principle of blinding.

### 2.4. Preparation of Pooled Protein Extracts

The tissues were carefully minced and thoroughly washed with wash buffer to remove residual blood. Protein extractions were performed following our previously described protocol [27]. Briefly, tissues were homogenized with stainless steel beads (1.4 mm) using a bead-beater (Bullet Blender, Next Advance, Troy, NY, USA), followed by sequential centrifugation steps to obtain a clear crude protein extract. Protein concentrations were determined using the Bradford assay (Bio-Rad, Hercules, CA, USA) and measured with a NanoDrop ND-1000 spectrophotometer (Thermo Scientific, Waltham, MA, USA). To minimize inter-sample variability, equal amounts of protein from each sample were pooled into a single tube for downstream analyses.

### 2.5. Tryptic Digestion and Mass Spectrometry-Based Protein Identification

Prior to LC-MS/MS analysis, proteins were digested into peptides using an in-solution tryptic digestion and guanidination kit (Thermo Fisher Scientific, Waltham, MA, USA), following the manufacturer’s instructions. The resulting peptides were analyzed using nanoLC-MS/MS (nLC-MS/MS- (Q Exactive™ HF, Thermo Fisher Scientific, Waltham, MA, USA)) employing a data-dependent acquisition method, in which the top 10 precursor ions were selected for MS/MS analysis within a scan range of 400–2000 *m*/*z*. Raw spectral data were processed using Proteome Discoverer SEQUEST software (version 2.2; Thermo Fisher Scientific, Waltham, MA, USA), and protein identification was performed against the Uniprot/SwissProt database to ensure high-confidence identifications [28].

### 2.6. In Silico Analysis of Proteomic Data

To investigate the biological significance of differentially expressed proteins, functional enrichment analyses were performed using Gene Ontology (GO), Kyoto Encyclopedia of Genes and Genomes (KEGG), Reactome (RKTM), and WikiPathways (WP) databases. Protein–protein interaction networks were constructed using the STRING database (https://string-db.org, accessed on 15 June 2025), with experimental evidence, curated databases, and co-expression data as active sources. Analyses were filtered with a false discovery rate (FDR) < 0.05 and a minimum gene count of 2 [29]. Visualizations of significantly enriched terms (*p* < 0.05) and interaction networks were generated as bitmap images and further refined using Adobe Illustrator (Version 6, Adobe Inc., San Jose, CA, USA). Additionally, bubble plots of enriched pathways and functional terms were created using SRPlot (ShenZhen University, Shenzhen, China) for clearer data representation.

### 2.7. Statistical Analysis

All statistical analyses were performed using GraphPad Prism, version 5.0 (Dotmatics, Boston, MA, USA). Multiple comparisons between groups were evaluated with two-way ANOVA and the unpaired *t*-test with Welch’s correction. A *p* value < 0.05 was considered statistically significant. For ordinal data involving two independent groups, the Mann–Whitney U test was used. DBP-treated and control groups were compared for four histological parameters: granulation tissue amount, inflammatory infiltrate level, collagen fiber orientation, and collagen pattern. In the study, the duration of DBP application to the flap model was treated as an independent variable, while inflammatory tissue surface area and protein expression differences (from nLC-MS/MS analyses) were treated as dependent variables. For nLC-MS/MS proteomic analysis, statistical evaluations were conducted using tools integrated with Proteome Discoverer 2.2.

## 3. Results

### 3.1. Photo Area Calculation Results

**Figure 1:**
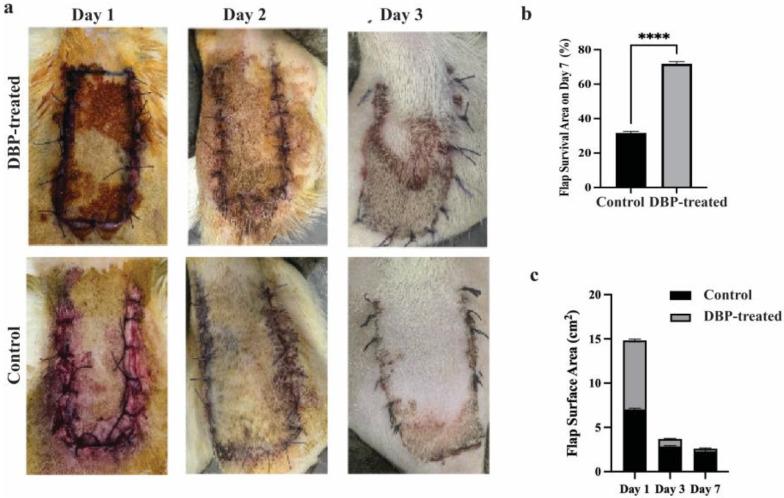
(**a**) Digital photographs of skin flap survival on postoperative days 1, 3 and 7 in the control and DBP-treated groups. (**b**) On the 7th day, the increase in flap survival rate in the boron group was found to be statistically significant. (*p* < 0.0001 ****). (**c**) The reduction and improvement in the total flap surface area on the 3rd and 7th days in the boron group were found to be statistically significant. (*p* < 0.0001).

Flap areas in photographs taken during the experiment were calculated with the imageJ program (Figure 1a). On day 7, the viable area/total area in the flap tissue in the control and boron groups was calculated as square meters. Flap survival rates were shown as %. Survival was calculated as 32.7% in the control group and 71.7% in the DBP-treated group. The increase in flap survival rate in the DBP-treated group was found to be statistically significant (*p* < 0.0001) (Figure 1b). Flap areas in the control and DBP-treated groups were recorded every day. Areas on days 1, 3 and 7 were statistically analyzed. The decrease and improvement in total flap surface area on days 3 and 7 in the boron group were found to be statistically significant (*p* < 0.0001) (Figure 1c).

### 3.2. Histopathological Evaluation

**Figure 2:**
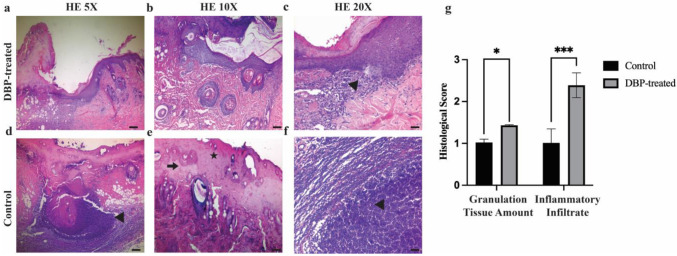
Histological analyses of wound healing. Representative hematoxylin and eosin (HE)–stained sections of skin flaps from control and DBP-treated rats seven days after treatment. (**a**–**c**) DBP-treated group showing well-organized granulation tissue and re-epithelialization at increasing magnifications (5×, 10×, 20×; scale bars = 200 µm, 100 µm, 50 µm, respectively). (**d**–**f**) Control group illustrating limited granulation and denser inflammatory infiltration. Arrowheads indicate inflammatory cell clusters with visible nuclei; arrows denote granulation tissue regions; stars mark collagen fiber deposition. (**g**) Quantitative histological scoring comparing granulation tissue amount and inflammatory infiltration between control and DBP-treated groups (* *p* < 0.05; *** *p* < 0.001).

**Figure 3:**
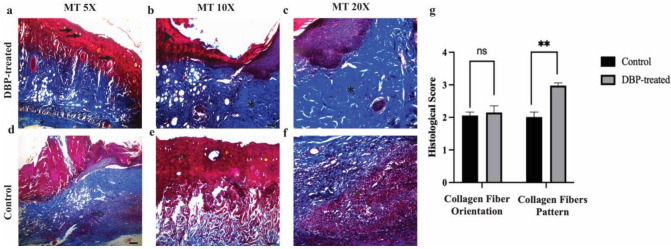
Histological analyses of collagen organization in wound healing. Representative Masson’s Trichrome (MT)–stained sections of skin flaps from control and DBP-treated rats seven days after treatment. (**a**–**c**) DBP-treated group showing enhanced granulation tissue, aligned collagen fiber bundles, and increased vascularization at increasing magnifications (5×, 10×, 20×; scale bars = 200 µm, 100 µm, 50 µm, respectively). (**d**–**f**) Control group displaying irregular collagen fiber orientation and weaker granulation tissue formation. Arrows indicate collagen fiber alignment; arrowheads denote vascular structures; asterisks mark dense collagen deposition zones. (**g**) Quantitative histological scoring of collagen fiber orientation and pattern between control and DBP-treated groups. No significant difference was observed in collagen fiber orientation (ns), whereas the collagen fiber pattern differed significantly between groups (** *p* < 0.01). In Masson’s Trichrome staining, collagen fibers appear blue, muscle and cytoplasm are stained red, and nuclei are dark purple.

Microscopic evaluation of the wounds was performed using flap excision specimens that included the entire wound area along with its margins. Hematoxylin and eosin staining, a standard method in dermatopathology, was applied to assess key wound healing parameters such as inflammatory cell infiltration, granulation tissue formation, initial fibrotic response, and epithelialization (Figure 2). Comparative histopathological analysis between the groups was conducted on day 7 post-injury. Granulation tissue score was significantly higher in the DBP-treated group (1.43 ± 0.76) compared to control (1.0 ± 0.0) (*p* = 0.034). Inflammatory cell infiltration score was significantly higher in the DBP-treated group (2.36 ± 1.5) compared to control (1.0 ± 0.0) (*p* = 0.003) (Figure 2e). To assess collagen deposition and extracellular matrix (ECM) remodeling, Masson’s Trichrome staining was employed, enabling both morphological and semi-quantitative evaluation of tissue architecture during wound repair (Figure 3). In the DBP-treated group, Masson’s Trichrome staining revealed intense blue coloration of collagen fibers, accompanied by an increased density of active capillary vessels, indicative of a vigorous and well-progressed proliferative phase. These histological findings suggest that SPP treatment may positively influence the wound healing trajectory. Collagen fiber orientation score was higher in the DBP-treated group (2.14 ± 0.53) compared to control (2.0 ± 0), not significantly (*p* > 0.05). Pattern of collagen score was significantly higher in the DBP-treated group (3.0 ± 1.04) compared to control (2.0 ± 0) (*p* = 0.003) (Figure 3e).

Further analysis demonstrated a statistically significant reduction in granulation tissue volume and inflammatory cell infiltration in the DBP-treated group compared to the controls. Additionally, collagen fibers exhibited a more organized and parallel fascicular arrangement, suggesting advanced tissue remodeling and structural maturation. Collectively, these results indicate that DBP application may accelerate the resolution of inflammation and enhance ECM reorganization, thereby facilitating more efficient and improved wound healing.

### 3.3. Label-Free Quantification and Comparative Proteome Analysis

To investigate the proteomic alterations induced by DBP treatment in the flap wound model, a label-free quantification approach was employed. Protein extracts from tissue samples were digested and analyzed using nHPLC-LC-MS/MS. The analysis led to the identification of a total of 1626 master proteins across all experimental groups. To increase confidence in the differential expression analysis, stringent filtering criteria were applied, including the presence of at least two unique peptides per protein, an abundance ratio above two, and a high-confidence false discovery rate (FDR). Following these filters, 179 differentially expressed proteins were identified between the DBP-treated group and the control. Among these, 14 proteins were found to be upregulated, while 165 proteins were downregulated in response to DBP treatment.

**Figure 4:**
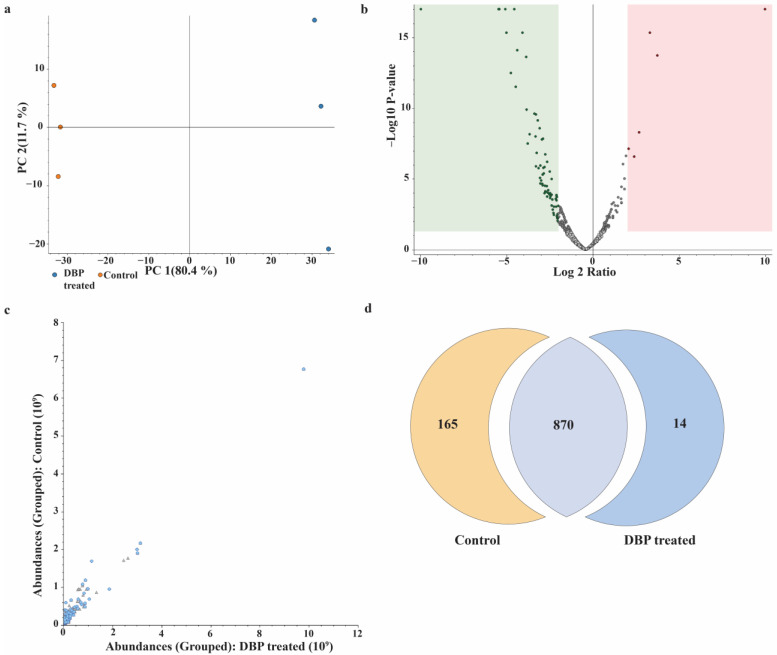
Label-free quantitative proteomic comparison between control and DBP-treated flap tissues. (**a**) Principal component analysis (PCA) illustrating clear separation between control and DBP-treated samples. (**b**) Volcano plot depicting differentially expressed proteins with a fold change threshold of ≥2 or ≤0.5 (*p* < 0.05). Upregulated proteins in the DBP-treated group are indicated in red, while downregulated proteins are shown in green. (**c**) Scatter plot showing the correlation of protein abundance between the two experimental groups. (**d**) Venn diagram displaying the distribution of identified proteins with ≥2 unique peptides. Among the 1626 identified proteins, 179 were differentially expressed, including 14 upregulated and 165 downregulated proteins in response to DBP treatment.

To elucidate the global proteomic alterations induced by DBP treatment, a series of comparative visualizations were generated to provide a comprehensive overview of protein expression profiles (Figure 4). Principal component analysis (PCA; Figure 4a) revealed a clear segregation between the control and DBP-treated groups, indicating distinct proteomic signatures. The volcano plot (Figure 4b) highlights significantly modulated proteins (fold change ≥2 or ≤0.5, *p* < 0.05), whereas the scatter plot (Figure 4c) demonstrates a strong correlation in overall protein abundance between samples, confirming dataset consistency. The Venn diagram (Figure 4d) illustrates the distribution and overlap of identified proteins (≥2 unique peptides) across experimental groups. Collectively, these complementary analyses emphasize the reproducibility and robustness of the quantitative proteomic differences elicited by DBP treatment.

### 3.4. Bioinformatics Analysis of Differentially Regulated Proteins

**Figure 5:**
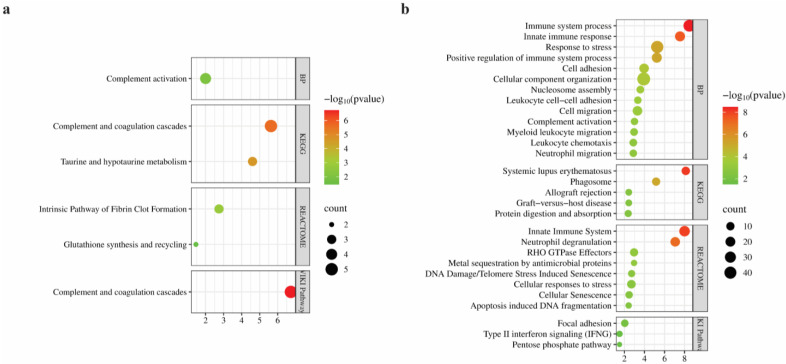
Bubble-plot visualization of pathway enrichment after DBP treatment. Pathway enrichment analysis was performed separately for (**a**) upregulated and (**b**) downregulated proteins, using multiple annotation databases: Biological Process (BP), Kyoto Encyclopedia of Genes and Genomes (KEGG), Reactome, and WikiPathways. Dot color indicates statistical significance, and dot size represents the number of proteins mapped to that pathway.

To elucidate the biological significance of the proteins that are differentially expressed in the DBP-treated group, comprehensive bioinformatics analyses were conducted, including GO, KEGG pathway, Reactome, and WikiPathways enrichment using the STRING database. The results were then visualized as bubble plots via SRPlot (Figure 5).

For upregulated proteins, GO biological process enrichment revealed a significant association with the “complement activation process”, which involved proteins such as Cfb, Serping1, Cfb-2, and Clr. KEGG pathway analysis highlighted two significantly enriched pathways: “Complement and coagulation cascades” (FDR: 2.37 × 10^−6^), and “Taurine and hypotaurine” metabolism (FDR: 2.49 × 10^−5^). Reactome pathway analysis further supported the involvement of upregulated proteins in the “Intrinsic pathway of fibrin clot formation”, involving Serping1, Klkb1, and A2m and “Glutathione synthesis and recycling”. Finally, WikiPathways analysis identified a highly significant enrichment in the “Complement and coagulation cascades pathway” (FDR: 1.86 × 10^−7^), with five matching proteins (Figure 5a).

**Figure 6:**
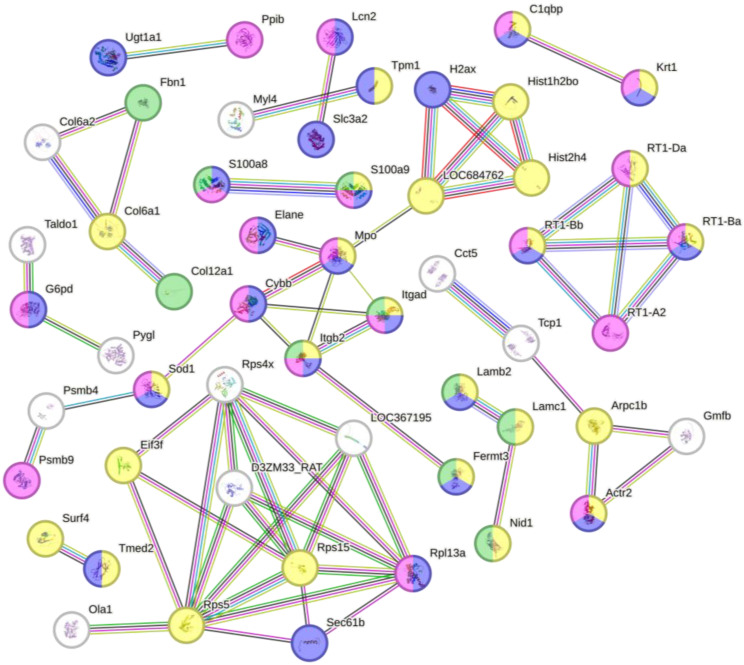
STRING protein–protein interaction network of downregulated proteins following DBP treatment. Nodes are color-coded by enriched GO Biological Process categories. Yellow: Cellular component organization (GO:0016043), Blue: Response to stress (GO:0006950), pink: Immune system process (GO:0002376), and green: Cell adhesion (GO:0007155). Each node represents a downregulated protein, with its gene name displayed on the node.

GO biological process enrichment of downregulated proteins revealed a strong association with immune-related processes. The term “immune system process” included 36 matching genes and showed a FDR of 3.33 × 10^−9^. Similarly, “innate immune response” (20 genes, FDR: 2.83 × 10^−8^) and “response to stress” (40 genes) were among the most significantly enriched categories. Additional enriched terms included “*cellular component organization*”, “complement activation”, “leukocyte chemotaxis”, “cell adhesion”, and “neutrophil migration” (Figure 6). KEGG pathway analysis identified “systemic lupus erythematosus” as the most significantly enriched pathway, including 10 proteins with roles in antigen presentation and chromatin structure. Additional enriched pathways included “phagosome”, “allograft rejection”, and “graft-versus-host disease”, each containing MHC class I and II-associated proteins such as RT1-Ba, RT1-Bb, RT1-Da, and RT1-A2. Other pathways such as “protein digestion and absorption” were also enriched, featuring structural and transporter proteins including Col6a1, Slc3a2, and Dpp4. Reactome analysis supported these findings with significant enrichment in the “innate immune system” pathway (24 proteins) and “neutrophil degranulation” (17 proteins). Several proteins were also involved in “metal sequestration by antimicrobial proteins”, including S100a8, S100a9, and Lcn2. Importantly, pathways associated with cellular damage and aging such as “DNA damage/telomere stress-induced senescence”, “cellular senescence”, and “apoptosis-induced DNA fragmentation” were enriched as well. Additional pathways such as “cellular responses to stress” and “RHO GTPase effectors” indicated links to cytoskeletal remodeling and stress signaling cascades. WikiPathways analysis revealed additional enriched terms such as “focal adhesion”, “type II interferon signaling (IFNG)”, and the “pentose phosphate pathway”, pointing to potential roles in cytoskeletal organization, oxidative balance, and immune regulation (Figure 5b).

## 4. Discussion

The present study demonstrated the therapeutic potential of a boron-containing hydrogel, namely DBP, in a flap wound model. Histological and proteomic findings indicated that DBP treatment enhanced tissue regeneration, collagen organization, and modulated immune pathways, resulting in improved healing compared to controls. Accelerated wound healing in DBP-treated flaps is notable, especially considering the ischemia-related challenges of flap models [30]. Given the scarcity of flap-specific boron studies, similar effects have been observed in excisional wounds, where boron stimulated fibroblast proliferation and reduced inflammation [14,31], paralleling our histological findings. Several research studies have reported regulation of matrix metalloproteinases and improved collagen synthesis by boron [32,33], which corresponds to the organized, parallel collagen bundles and advanced extracellular matrix remodeling we observed. Sedighi Pirsaraei et al. highlighted boron’s antioxidant, antimicrobial, and pro-angiogenic properties [12], supported by the presence of active capillaries in our samples. Boron hydrogels used in diabetic and burn wound models have similarly promoted angiogenesis, collagen deposition, and reduced inflammation [13,34,35]. Demirci et al., for instance reported increased fibroblast activity and VEGF expression with boron hydrogel treatment [13], which mirrors our histological evidence of enhanced vascularization and immune regulation. A randomized controlled trial showed that sodium pentaborate gel significantly improved healing rates and reduced recurrence in diabetic foot ulcers [24]. Furtermore, Doğan et al. reported that sodium pentaborate–pluronic hydrogel enhanced fibroblast migration, antioxidant enzyme activity, VEGF and TGF-β expression, wound contraction, and collagen deposition in vitro and in vivo [23].

In ischemic random-pattern flap models, treatments like deferoxamine (DFO) and vascular endothelial growth factor (VEGF) have improved capillary density, tissue perfusion, and flap survival [6,36,37]. Wang et al. reported that local DFO injections in diabetic mouse flaps increased HIF-1α, VEGF expression, and capillary formation, enhancing flap viability [6]. Similarly, VEGF therapy reduced distal necrosis in ischemic flaps [37]. Our histological findings those marked by active capillary proliferation reflect similar angiogenic benefits. Additionally, therapies involving stem cell–based scaffolds and antioxidants have shown to reduce inflammation and support extracellular matrix integrity in flap models [38,39,40]. Our data reveal comparable outcomes, with significantly reduced inflammatory cell presence and well-organized collagen architecture in the DBP-treated group. These similarities suggest that our boron-containing hydrogel, much like DFO or VEGF-based approaches, effectively addresses the flap ischemia hallmarks such as perfusion deficits, inflammation, and poor matrix remodeling. It provides benefits similar to more advanced bioactive treatments while offering the ease and precision of a simple topical application.

To further elucidate the molecular mechanisms of DBP treatment, we performed a label-free proteomic analysis comparing treated and control flap tissues. This analysis revealed a total of 179 differentially expressed proteins, among which only 14 were upregulated, while a striking 165 proteins were downregulated in response to DBP treatment. This large difference suggests that boron treatment may act mainly by suppressing excessive protein activity, particularly in pathways linked to inflammation and stress. Rather than triggering broad activation, boron seems to create a more controlled and balanced tissue environment, which aligns with the reduced inflammation and improved tissue structure we observed histologically.

Bioinformatic analysis of upregulated proteins revealed enrichment in complement and coagulation cascade components. Activation of these pathways likely accelerates hemostasis and microbial clearance, both essential in early wound stabilization. Notably, the elevation of Complement Factor B (Cfb) and Serping1 points to activation of the alternative complement pathway, which aids opsonization, immune modulation, and leukocyte recruitment. In wound healing contexts, Factor B contributes to C3 convertase formation, supporting debris clearance and inflammation regulation [41]. Serping1 also regulates fibrin turnover, preventing excessive clotting and chronic inflammation [42]. The observed coordinated upregulation of both complement and coagulation proteins suggests a tightly regulated hemostatic-immunological interface in DBP-treated wounds. This dual activation secures hemostasis, supports microbe defense, and ensures a controlled inflammatory response, features essential for swift and scar-minimized healing. Recent biomaterials have shown that controlled activation of these cascades can reduce infection risk and improve tissue integration. For instance, chitosan-based hydrogels enhanced coagulation through complement activation while maintaining biocompatibility, accelerating healing in vivo [43]. Similarly, biomaterial design that triggers mild complement and platelet activation facilitates balanced inflammation and angiogenesis [44].

DBP treatment activated taurine and hypotaurine metabolic pathways, known for antioxidant and anti-inflammatory effects. Taurine, a sulfur-containing osmolyte and antioxidant, enhances wound tensile strength, limits mast cell degranulation, and promotes angiogenesis and collagen organization [45]. Furthermore, enrichment of glutathione synthesis and recycling pathways point to elevated antioxidant defenses. Glutathione, a central molecule in cellular defense against ROS, has been shown to reduce fibrosis and expedite wound resolution when incorporated into biomaterials [46]. The activation of these pathways under DBP treatment likely contribute to the controlled inflammation and organized matrix structure observed on day seven. Together, the upregulated protein profile suggests that our boron-containing hydrogel enhances early coagulation, strengthens antioxidant defenses, and supports immune–coagulation crosstalk crucial for infection control and tissue repair.

Proteomic analysis showed broad suppression of immune-related proteins, including those involved in leukocyte chemotaxis, neutrophil migration, and degranulation. Attenuation of these processes has been linked to improved regeneration and reduced fibrosis in biomaterial-based wound models [47]. Proteomic analysis revealed significant downregulation of S100A8, S100A9, and Lcn2, key regulators of metal sequestration in nutritional immunity. These proteins normally inhibit microbial growth by binding zinc and iron [48]. Prolonged expression of S100A8/A9 can delay healing by disrupting matrix remodeling, especially in chronic wounds [49]. Furthermore, downregulation of S100A8/A9 has also been shown to enhance angiogenesis and endothelial survival in myocardial and cutaneous injury models [50,51]. Therefore, the observed downregulation of metal-sequestering proteins in DBP-treated flaps likely reflects a more stabilized vascular microenvironment and a shift from antimicrobial defense mechanisms toward controlled tissue repair and neovascularization.

Key antigen-presentation proteins, including MHC class I and II molecules such as RT1- Ba, Bb, Da, and A2, were significantly downregulated, along with phagosome-related proteins and pathways linked to allograft rejection and autoimmune responses. This suggests a shift from immune surveillance to tissue repair. MHC II expression typically peaks early in wound healing but declines as regeneration proceeds state [52]. Persistent MHC II activity, as seen in chronic inflammatory states, can impair repair by sustaining fibroblast activation and immune cell recruitment [53]. In flap ischemia–reperfusion, excessive antigen presentation may worsen inflammation and hinder revascularization; its suppression in DBP-treated flaps likely supports more efficient remodeling and reduced fibrosis.

Proteomic analysis showed significant downregulation of proteins involved in DNA damage response, senescence, and apoptosis, suggesting that boron hydrogel helps reduce genotoxic stress in flap tissue. Ischemia–reperfusion is known to cause oxidative DNA damage via pathways like p53 and γ-H2AX, which can lead to cell death and fibrosis [54,55]. Suppressing these signals has been shown to enhance epithelial survival and angiogenesis in flap models, while antioxidant or senolytic biomaterials improve healing by limiting senescence [56]. Thus, the observed downregulation of stress and senescence-associated proteins in the DBP-treated group likely reflects a protective cellular response that limits oxidative injury, preserves tissue viability, and promotes regenerative remodeling.

Downregulation of proteins governing focal adhesion, RHO GTPase effectors, and cell adhesion also emerged. Such reduction is characteristic of healing microenvironments. This modulation facilitates cell migration by reducing excessive cell–matrix interactions that could otherwise hinder re-epithelialization. Recent studies demonstrate that Rho GTPases such as RhoA and Rac1 are critical for fibroblast spreading and mechanosensitive migration in 3D hydrogel systems, where precise regulation enables efficient cell guidance and matrix remodeling [57]. Local regulation of RhoA activity through mechanosensitive has been shown to promote focal adhesion turnover and facilitate cell migration in fibrous environments [58]. Collectively, these findings support the view that controlled suppression of Rho-mediated adhesion dynamics is a key mechanism in promoting efficient wound regeneration.

Lastly, proteomic analysis revealed decreased activity in IFN-γ signaling and the pentose phosphate pathway. These are associated with a shift from inflammatory and metabolic stress toward resolution and controlled repair. Chronic IFN-γ signaling has been shown to impair wound healing, with studies demonstrating that IFN-γ deficiency accelerates wound closure, enhances angiogenesis, and increases collagen deposition [59]. Similarly, IFN-γ activity was shown to inhibit fibroblast proliferation and migration, thereby reducing fibrotic remodeling in models of epidural and arthrofibrosis [60,61]. This suggests that a decrease in IFN-γ activity, as seen in DBP-treated flaps, may alleviate excessive immune signaling and fibrosis. Taken together, the downregulation of the diverse immune, stress, and structural pathways suggests that our boron-containing hydrogel fosters a controlled molecular environment that minimizes inflammatory burden, preserves cellular integrity, and enables coordinated tissue remodeling, key requirements for successful flap wound healing.

Although sample size in the study was limited, the use of standardized surgical procedures and pooled proteomic analysis minimized biological variability, making the present findings reliable within the scope of an exploratory investigation. The study did not explore a dose–response gradient nor include a universal positive control, as the objective was to validate the efficacy of a literature-supported SPP-containing formulation in a flap ischemia model. Future work will incorporate multiple SPP concentrations and an active comparator to quantify relative efficacy.

## 5. Conclusions

This study highlights the significance of short-term improvements observed with DBP in a rat ischemic flap model. In a single model with a 7-day observation period, DBP increased viable flap area, improved histological readouts such as angiogenesis, inflammation, and collagen organization, and was associated with proteomic changes consistent with lower immune and stress signaling. These results support short-term efficacy in this preclinical setting. Because we did not assess long-term outcomes, chronic or infected wounds, or compare DBP with established treatments, the implications should be interpreted within these limits. DBP is a promising candidate for further evaluation in extended and comparative models to determine durability, safety, and relative benefit.

## Figures and Tables

**Table 1:** Histological parameters used to assess and calculate wound healing state.

Histological Parameters	Scoring System	Histological Grading
Amount of granulation tissue	Profound	1
	Moderate	2
	Scanty	3
	Absent	4
Inflammatory infiltrate	Plenty	1
	Moderate	2
	A few	4
Collagen fiber orientation	Vertical	1
	Mixed	2
	Horizontal	4
Pattern of collagen	Reticular	1
	Mixed	2
	Fascicle	4

## Data Availability

The datasets generated during the current study are available from the corresponding author on reasonable request.

## Abbreviations

The following abbreviations are used in this manuscript:

DBP	Dermobor Plus
SPP	Sodium pentaborate pentahydrate
VEGF	Vascular endothelial growth factor
ECM	Extracellular matrix
MT	Masson’s Trichrome-stained
HE	Hematoxylin and eosin
FDR	False discovery rate
GO	Gene Ontology
DFO	Deferoxamine
nLC-MS/MS	nanoLC-MS/MS
KEGG	Kyoto Encyclopedia of Genes and Genomes
WP	WikiPathways
